# Compression-Aware Aggregation and Energy-Aware Routing in IoT–Fog-Enabled Forest Environment

**DOI:** 10.3390/s21134591

**Published:** 2021-07-04

**Authors:** Srividhya Swaminathan, Suresh Sankaranarayanan, Sergei Kozlov, Joel J. P. C. Rodrigues

**Affiliations:** 1 Department of Information Technology, SRM Institute of Science and Technology, Chengalpattu 603203, Tamil Nadu, India; srividhs1@srmist.edu.in; 2 ITMO University, 197101 St. Petersburg, Russia; kozlov@mail.ifmo.ru (S.K.); joeljr@ieee.org (J.J.P.C.R.); 3 Federal University of Piauí (UFPI), Teresina 64049-550, PI, Brazil; 4 Instituto de Telecomunicações, 6201-001 Covilhã, Portugal

**Keywords:** forest monitoring, Internet of Things, fog, LLN, aggregator

## Abstract

Forest fire monitoring is very much needed for protecting the forest from any kind of disaster or anomaly leading to the destruction of the forest. Now, with the advent of Internet of Things (IoT), a good amount of research has been done on energy consumption, coverage, and other issues. These works did not focus on forest fire management. The IoT-enabled environment is made up of low power lossy networks (LLNs). For improving the performance of routing protocol in forest fire management, energy-efficient routing protocol for low power lossy networks (E-RPL) was developed where residual power was used as an objective function towards calculating the rank of the parent node to form the destination-oriented directed acyclic graph (DODAG). The challenge in E-RPL is the scalability of the network resulting in a long end-to-end delay and less packet delivery. Additionally, the energy of sensor nodes increased with different transmission range. So, for obviating the above-mentioned drawbacks in E-RPL, compressed data aggregation and energy-based RPL routing (CAA-ERPL) is proposed. The CAA-ERPL is compared with E-RPL, and the performance is analyzed resulting in reduced packet transfer delay, less energy consumption, and increased packet delivery ratio for 10, 20, 30, 40, and 50 nodes. This has been evaluated using a Contiki Cooja simulator.

## 1. Introduction

Forest or wildfire has a huge impact on environmental losses that damage the flora and fauna. These fires happen due to seasonal variations such as high temperature, high humidity, and so forth. Every year all over the world, the percentage of forest fire is increasing. There have been a lot of developments made in the field of wildfire fighting regarding disaster response such as early warning systems and real-time exchange of data for forest fire monitoring. Recent developments in information and communication technology are already having a huge impact, specifically in forest fire detection systems [1].

Internet of Things (IoT) is the latest buzzword that allows objects or devices to be connected wirelessly to the internet. Mostly, the devices deployed in the remote area are resource constrained. These devices also called sensor nodes are deployed in a remote area that is constrained in terms of processing power, power allocation, and radio resources. Due to the drastic increase in the evolution of smart devices, many remote-based applications, such as military surveillance, smart agriculture, and forest management, need a supportable solution for power-constrained environments [2].

In most IoT-based applications [3], energy is very scarce. In applications such as forest fire where there is no power backup, energy conservation is a big challenge where regular monitoring is required for preventing forest fires. There is a lot of research going on in terms of routing, preserving the energy of the node states, redundancy-based routing, clustering, compressed routing, optimized duty cycling [4], latency [5], etc. Most of the existing protocols and approaches are targeted towards improving the network lifetime. The IoT-enabled environment is made up of low power and lossy networks [6]. Low Power and Lossy Networks (LLNs) are collectively made of sensor nodes with limited energy, computing capacity, and memory storage. These constraints form the network communication with packet loss, lower data transmission rates, disconnectivity in the network, etc. Applications specific to environment monitoring need development by the way of better-enhanced routing in LLNs. Recently, the development of the Internet of Things makes its application more extensive; the scope of application extended to the location [7], vehicle networking [8], and other related fields.

Currently, researchers concentrate on routing [9,10,11], energy-based clustering [12], Medium Access Control (MAC)-oriented protocols [13,14,15], and related issues. To improve the performance of the routing protocol in forest fire management, a power-saving routing is needed to increase the overall network lifetime. The routing protocol for low power lossy networks (RPL) is proposed as an IPv6 routing protocol for LLNs by the ROLL Working Group in the Internet Engineering Task Force (IETF). RPL is intended to support User Datagram Protocol (UDP) communication for a proper tradeoff between power consumption and timely communication.

Energy is limited to routing in forest fire. Therefore, the conservation of energy of the sensor nodes is a significant challenge in the real-time scenario. Routing plays a vital role to optimize the utilization of nodes’ energy. The objective function used in routing protocol for low power lossy network (RPL) rank calculation usually depends on hop count. In energy-efficient RPL (E-RPL) [16], the objective function is the residual power (RP) of the node to calculate the rank of the parent node. The highest rank node is assigned as the parent node in the Destination Oriented Directed Acyclic Graph (DODAG) tree. However, energy is not the only parameter to be included. The optimized root id is framed by considering the parameters such as scalability, the distance between the nodes, transmission range, etc. The energy-efficient RPL is validated using the Contiki Cooja simulator. The challenge in energy-efficient RPL (E-RPL) is the scalability of nodes. When nodes are increased in the network and randomly deployed with partial nodes in the interference range, the network experiences a long delay in communicating to the border router. In time-sensitive applications such as forest fire where data are critical, it is very much needed in sending the data with less delay and more packet delivery ratio for real-time analysis. Additionally, the energy consumption of sensor nodes in energy-efficient RPL increases with a varying number of nodes for different transmission range.

So, towards obviating the issue of minimal delay and packet delivery ratio with reduced power consumption in forest fire networks, we have proposed here a compression-aware aggregation-based approach for energy-efficient RPL. The aggregation scheme has been proposed in energy-efficient RPL for balancing the load, minimizing the delay, improving the scalability of the network, and reducing the power consumption. In addition to aggregation, data compression is also included at the aggregator for reducing the number of packets to be transmitted to the border router and, in turn, optimize the power consumption. The proposed work is implemented in Contiki OS with a Cooja simulator. The major contributions of the paper are as follows:

- Region formation and aggregator selection in CAA-ERPL.
- Data aggregation with data compression at the aggregator.
- Data reconstruction at the border router.
- Comparative analysis of the performance of CAA-ERPL versus E-RPL.

The paper is organized in the following way. Section 2 gives a detailed review of related research works in RPL routing in IoT. Section 3 explains the energy-based RPL routing for the forest environment. Section 4 details the proposed compression-based data aggregation scheme. Section 5 provides the simulation setup, results, and discussions on CAA-ERPL. Section 6 provides the concluding remarks and future work.

## 2. Related Works

In this section, the various techniques related to IoT-based routing in energy constrained environment are discussed.

Homaei et al. proposed an enhanced model for data aggregation for IoT environments [17]. In the RPL-based routing, the number of child nodes under a parent decides the load of the entire network. Sometimes, if the child nodes are more for a single parent, it will create congestion towards connecting the parent node. Dynamic aggregation of data is done at the parent node using learning automata. Every node in the network has its own learning automata (LA), which does the operation of aggregation and forwarding data. Learning automata RPL (LA-RPL) is implemented using a Contiki Cooja simulator. The challenge in this work is network congestion due to the absence of a trickle timer for synchronization.

The authors in this work proposed a resource-constrained routing path by utilizing an aggregator node [18]. The routing protocol RPL changes its route periodically based on available resources. Dynamic routing has been proposed where the DODAG formation is fast, and the sink node collects the data from the lower layer nodes. The objective function of adaptive RPL (A-RPL) is decided based on the environmental behavior and network overload parameters. The challenge in this work is also the absence of a dynamic trickle timer and the congestion of aggregated packets during the forwarding process.

Conti et al. suggested a group-based routing to improve the performance of the RPL. Multi-cast routing is done among the sensor nodes from each cluster. Since clustering and combining data packets are done at the parent node, network load is reduced. In addition, the parent node is chosen in a way that is capable of performing group communication [19]. The main improvement needed in this work is that energy is not a metric for selecting a parent node.

Taghizadeh et al. [20] proposed an improved metric for calculating the rank of the destination-oriented directed acyclic graph (DODAG). The rank of the node is determined based on the context of the respective sensor node. Since it depends on context awareness, packet loss is gradually reduced in the routing protocol. The drawback of this work is that it has taken only the context for the calculation of the RANK metric in the objective function. None of the link-oriented metrics are considered in this work that will have more impact on the network load.

In [21], the authors recommended two different MAC-based metrics for routing. First, R-metric improves the standard expected transmission range objective function (ETX-OF) by taking packet drops at each node into account. The next Q-metric will manage the load of the network by considering the number of packets involved in transmission and reception. Hence, an optimized modified routing for RPL is the solution for the challenges listed above for forest fire. This work has combined both ETX and load metrics to strengthen the routing and compared the performance on delay constraints versus reliability constraints. The challenge in this work is that the scalability of the network is not taken for the performance of the work.

Different data-centric routing algorithms are proposed for IoT. Content centric routing for RPL (CCRPL) is proposed in [22]. Whenever the content is available at the routing node, it will consolidate and forward the data towards the destination. Data aggregation is done at the immediate node before forwarding. CCRPL is compared with traditional RPL in terms of metrics such as reliability, latency, power efficiency, etc. Though aggregation is incorporated here, properties and efficiency on aggregation are not specified, which is a major drawback.

Cluster-based RPL (C-RPL) was implemented by Zhao et al. for IoT in the real-time environment [23]. C-RPL introduced a forwarding scheme with optimal selection of parent nodes towards reducing the delay in the communication between source and destination. For data transfer, scheduling based on priority is integrated into RPL. Simulations were done using NS3 and C-RPL performed better than RPL. The challenge in this work is that the overall packet transmission time was affected in terms of packet loss in C-RPL.

Multiple unequal cluster-based routing is done for LLN [24]. The entire network is divided into multiple clusters of unequal sizes. The residual energy of the node is taken for choosing the cluster head. Data aggregation is done at the cluster head, and finally, it is transmitted to sink. There are some time delays in the formation of clusters initially. Simulations are done using the COOJA simulator, and the performance of the protocol is compared with RPL in terms of network lifetime and scalability. The main challenge found in this work is that the power taken for cluster formation and delay in the control message transfer have not been analyzed.

In [25], the authors detailed the need for aggregation in WSN. An elaborated survey with analysis is done for aggregations and lists the advantages of aggregation. Aggregation minimizes the size and amount of data transfer. Few challenges related to aggregation, such as limited communication based on an interval basis, data compression rate, energy optimization, choosing aggregation based on application scenarios, etc., are summarized by the authors.

In [26], authors have proposed an aggregator-based RPL for IoT–Fog-based power distribution system. The authors here have improvised the Contiki RPL by introducing the concept of an aggregator among smart meters based on ETX as an objective function. The aggregator-based RPL is under the assumption that energy meters are connected to power, and so, energy constraint is not taken into consideration. The challenge in this work is that other metrics such as CPU utilization, number of parent changes, and Qos metric are not taken into consideration in this work. Additionally, only ETX was taken for aggregator selection in forming the DODAG, and other metrics were not considered.

In [27], authors have developed an energy-aware routing protocol (EAGDA-RPL) using grid-based data aggregation scheme. This has been developed for agricultural IoT applications. The routing protocol here forms equal-sized grid on a square network to reduce congestion among grid head nodes. Additionally, grid head is selected on a rotational basis. Each grid head uses ETX as a metric for selecting the optimal parent for data transmission. The challenge in this work is that energy is not taken into consideration for DODAG formation with grid head, where these sensor nodes are resource constrained. Additionally, the unequal grid size is not taken into consideration, which is another challenge.

From the related works, it is observed that some improvements have been carried out on RPL towards extending the network lifetime and improving the packet delivery ratio and reliability. Though there are improvements, the role of aggregation is not considered in RPL with energy as an objective function in the previous work, which is one of the main drawbacks. There are also other limitations in the previous work on RPL such as energy consumption, predicting parent node, time taken for forming DODAG structure, etc. These works were not focused on forest fire application, in particular, where energy constraint is a big challenge. These limitations have motivated the proposed work by involving the aggregator with energy as an objective function and data compression in RPL to balance the network load and reduce the number of nodes to be involved for the DODAG structure. This has resulted in an increased packet delivery ratio, reduced power consumption, and less end-to-end delay. The details are explained in the forthcoming section.

## 3. Energy-Based RPL Routing for IoT–Fog-Enabled Forest Region

For routing the data in IoT–Fog-enabled forest fire network, the most important criteria is energy-efficient routing with minimal delay and timely delivery. The reason being forest fire monitoring requires timely information for real-time analysis and quick action. This requires the need to propose an energy-efficient routing for IoT–Fog-based environment for forest fire networks. So, before going into the details of the proposed work, we talk in brief about Contiki RPL, which is the most predominant routing protocol in the IoT environment. Following that, we talk about energy-efficient RPL, which is the modification of Contiki RPL and compression-aware aggregation-based RPL. These details are explained below.

### 3.1. Contiki—RPL

RPL [28] construction comprises destination-oriented directed acyclic graph (DODAG) discovery, formation, and monitoring. RPL is formed with the exchange of ICMPv6 control messages for route formation. These control messages help for exchanging the DODAG information and, finally, forms a routing topology. Every node first sends the DODAG information solicitation message (DIS) to enquire about its neighborhood. Destination advertisement object (DAO) is used to identify and send the control message in the reverse direction to mark the visited nodes towards the root.

Every node position is fixed concerning the DODAG root node. The rank of the node increases towards the leaf and decreases towards the root. Rank 0 node acts as the root node. The rank of the node is calculated using an objective function. The formation of topology starts from the root node. First, the root sends the DODAG information object (DIO) message to all the neighbor nodes, which will receive and process the same. The process of broadcasting the DIO to the one-hop neighbors is repeated until the leaf nodes.

Once any of the one hop nodes join the DODAG, it will extend the path of routing towards the DODAG root. All the intermediate nodes calculate their RANK in DODAG and share their rank with the root node. If any of the nodes are not in the transmission range, it will send the DIS message at regular intervals to its neighbors. The process is repeated until all the nodes join the DODAG.

### 3.2. Energy-Based Routing Protocol for Low Power Lossy Networks (E-RPL)

The proposed idea for forest fire monitoring is that all the sensor nodes are randomly deployed. For RPL communication, DODAG has to be formed first. All the nodes select their parent from the list based on the RANK, which is calculated based on the objective function. For E-RPL, the objective function is the residual power (RP) of the node. Though residual energy plays an important role in the energy-constrained environment, it also needs an expected transmission range (ETX) to improve the performance. In E-RPL, both ETX and residual energy are used as an objective function in validating the network performance in terms of delay, packet delivery ratio (PDR), and CPU utilization for varying network sizes of 10, 20, 30, 40, and 50 nodes.

When all the sensor nodes send the data directly to the border router via RPL routing, it takes more time to reach the destination. Specifically, if the number of nodes is increased, it results in an increased end-to-end delay and less PDR. Additionally, the total energy consumption of the network increases with a varying number of nodes for reaching the border router or Fog node where real-time analysis is done. The level of nodes in the RPL DODAG ROOT increases when scalability increases. Though an optimized rank calculation is done with expected transmission range (ETX) and energy to reduce the size of DODAG in the entire network, the role of the aggregator is highly appreciated.

In the proposed work, instead of routing the data packets from all nodes to Fog nodes using E-RPL, aggregation and data compression are carried out at the aggregator for every region in the network area using E-RPL, where energy and ETX are applied to all aggregators, which will improve the performance of the network in terms of PDR and less overall energy consumption.

## 4. Aggregation-Based Compressed Sensing

In traditional wireless sensor networks, cluster heads act as aggregators for cluster-based routing. The formation of cluster head in the wireless sensor network is based on the Time Division Multiple Access (TDMA) scheme as followed in the Low Energy Adpative Clustering Hierarchy (LEACH) protocol. However, in terms of an IoT-based routing protocol where 6lowpan communication is used, RPL routing protocol is used. In RPL, the DODAG structure is used for routing among the 6lowpan sensor nodes, which are unique as compared to traditional sensor routing. The novelty of our work is introducing the concept of an aggregator in RPL using the energy as objective function, which is different from the cluster formation in LEACH routing protocol in sensor networks. Secondly, data compression is widely used in sensor routing. However, such data compression for reducing the redundant data transmission with reduced power consumption is not introduced in aggregator in RPL routing for forwarding to the border router. So, towards reducing the dimension of data, redundant data, loss of data, and the number of transmissions, compressed sensing is introduced in aggregator in RPL routing protocol, which is the second novelty. This makes the compressed sensing-based data aggregation novel and unique in RPL-based routing resulting in optimized and energy-efficient routing in the IoT-based environment. Details on aggregator selection and compressed sensing are discussed below.

### 4.1. Region Formation

**Figure 1:**
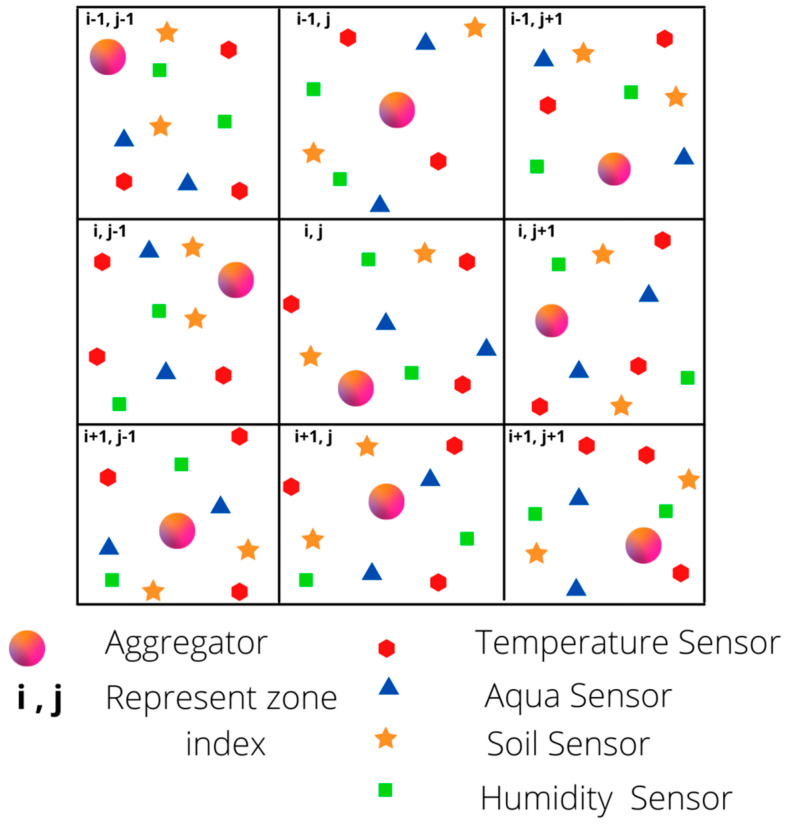
Network Model.

The entire forest area consists of “M” nodes that sense various parameters such as temperature, humidity, soil moisture, oxygen level, carbon dioxide level, rainfall, etc. The area is divided into several regions based on the energy resource of the aggregator node. An aggregator node is placed for every region, which is responsible for aggregating the sensed data based on its type. The aggregator node act as a collector for each split region. All the sensor nodes lie at leaf level in the DODAG structure. The aggregator nodes act as an intermediate parent, which collects group aggregate using compressed sensing and forwards the data towards the root of DODAG. The selection of the parent node is carried out with the aggregator constraint as ETX and power level. Figure 1 shows the network representation of random nodes and aggregator nodes.

### 4.2. Aggregator Selection

Data collection and aggregation at intermediate nodes for efficient transmission have to be considered in the network design itself. In the proposed work, data aggregation plays an effective role in compressing the sensor data before transmitting it towards the border router. The aggregator in each region is selected by the following Algorithm 1.
**Algorithm 1:** Aggregator Selection.**Input:** Number of regions, set of nodes in the region**Output:** Optimal aggregator node1. Initialization stage:a. Calculate the total power level of all sensors in the region.b. Calculate the individual power level of all sensor nodes.c. Calculate the probability of percentage for every node to become an aggregator.2. Steady phase    The node with higher value of probability becomes an aggregator for that region.

### 4.3. Data Aggregations

Heterogeneous data aggregation acts as a vital role in wireless sensor routing [29] especially in forest monitoring systems [30]. Specific to the forest region, several sensor nodes are deployed randomly in the region, which may sense the same value of data for a limited time. In such cases, data redundancy and many data retransmissions occur, which would deplete the resources. Data aggregation will remove the data duplications and group the data based on its type, then, forward it to the root.

#### 4.3.1. Formation of a Sparse Data Matrix

As per the network model in the proposed framework, the forest region is divided into “N” regions. These regions are not of equal size, since the nodes are randomly deployed in the hilly region. For each region, an aggregator is chosen based on its transmission range. If there are “N” regions, there will be “N” aggregators. The representation for an nth aggregator is specified by Agg_n_ where n ϵ ϕ Agg. Agg_n_ gathers the sensor data from the deployed heterogeneous sensors in its region. Assume that there are “m” nodes in the region “n” and “ith” node senses the environmental data d_ni_ and transmits to the Agg_n_ where (0 ≤ i ≤ m)M.

The sensor data are generated and transmitted with some specified limited interval time. For a time period “t”, the data sent by the sensors to the aggregator node Agg_n_ in the region are given by Equation (1).

(1) $$D_{n}={\left[d_{n1},\;d_{n2},\;d_{n3},\ldots \ldots \ldots \ldots d_{nM}\right]}^{T}$$

where

$$D_{n}\;\in \;\mathbb{R}\left(M\;\times \;1\right)$$

$D_{n}$ is M-dimensional real-time data. The data collected from the sensors with respect to time are not sparse at all. The sparse basis is calculated for the sensed data as given in Equation (2).

(2) $$D_{n}=I_{n}{\;\times \;\delta }_{n}$$

$${\;D}_{n}=\begin{array}{ccc} {}[{\;I}_{n1} & I_{n2}\;\ldots \ldots & I_{\mathrm{nM}}\;] \end{array}[\begin{array}{ccc} \delta_{n1} & \delta_{n2}\ldots \ldots \ldots & \delta_{\mathrm{nN}} \end{array}\;]{\;}^{T}$$

where

$${\;\delta }_{n}=I_{n}{}^{T}\;\times \;D_{n}$$

#### 4.3.2. Aggregator Compression Matrix

After the data $D_{n}\;\epsilon \;\mathbb{R}\;\left(M\;\times \;1\right)$ are specified by sparse basis $\delta_{n}\;\epsilon \;\mathbb{R}\;\left(M*M\right)$, it is now compressed as given in Equation (3). The use of this observation matrix is to reduce the dimension of data collected at the aggregator and also reduce the loss of information. The flow of aggregation is depicted in Algorithm 2.

$${\;D}_{n}\;\epsilon \;\mathbb{R}\;\left(M\;\times \;1\right)\to {\;\mathrm{CD}}_{n}\;\epsilon \;\mathbb{R}\;\left(N\;\times \;1\right)$$

$${\mathrm{CD}}_{n}=\phi_{n}\;\times \;D_{n}$$

(3) $${\mathrm{CD}}_{n}=I_{n}{\times \delta }_{n}\times \phi_{n}$$

**Algorithm 2:** Sensor data aggregation.**Input:** D_n_—data from environmental sensor **Output:** Compressed data ${\mathrm{CD}}_{n}$
 1. Compute sparse matrix for sensor data collected 
$D_{n}=I_{n}\times \delta_{n}$
  Where $\delta_{n}=I_{n}{}^{T}\times D_{n}$
 2. Compute aggregator compression matrix 
${\mathrm{CD}}_{n}=\phi_{n}\times D_{n}$
 
${\mathrm{CD}}_{n}=I_{n}\times \delta_{n}\times \phi_{n}$
 3. **Return** Aggregator compressed data ${\mathrm{CD}}_{n}$


#### 4.3.3. Data Reconstruction at the Border Router

The compressed data [31] from various aggregators are reconstructed at the border router using Equation (4).

(4) $$RD_{n}=\mathrm{argmin}\;∥D_{n}∥\;l0{\;\mathrm{subject}\;\mathrm{to}\;\mathrm{CD}}_{n}=D_{n}\times \phi_{n}$$

### 4.4. Energy-Efficient RPL Routing for Aggregators

The proposed idea for forest fire monitoring is that all the sensor nodes are randomly deployed. For RPL communication, DODAG has to be formed first. All the nodes select their parent from the list based on the RANK, which is calculated by the objective function. For energy-efficient RPL (E-RPL) [16], the objective function is the residual power (P) of the node. The proposed method is represented as a mathematical model. Assume that the RPL route looks like a graph. Graph G is made of vertices “v” called aggregators and edges “e” called links. The set of all vertices are the number of aggregators in the network. Agg = {Agg_1_, Agg_2_, Agg_3_, …, Agg_n_}. The steps for E-RPL are given in Algorithm 3.
**Algorithm 3:** DODAG aggregator parent selection.**Input:** Set of all aggregators $\left({\mathrm{Agg}}_{1},{\mathrm{Agg}}_{2},\ldots ,{\mathrm{Agg}}_{n}\right),$ root node—RN, initial rank—unknown, PN- parent _node**Output:** Optimal aggregator to act as a parent 1. **For** all vertices ${\mathrm{Agg}}_{i}\;€\;\left\{\mathrm{Agg}\right\}$  a. Update the value of power level RP for each vertex in V  b. Calculate power of each aggregator $P\left({\mathrm{Agg}}_{i}\right)$ and ETX of each region    
$P\left({\mathrm{Agg}}_{i}\right)=\frac{\left(\mathrm{Initial}\_\mathrm{power}\;–\;\mathrm{Consumed}\_\mathrm{power}\;\right)\;}{\mathrm{Initial}\_\mathrm{power}}\;$

       
$\mathrm{ETX}\;\left(\mathrm{Agg}\left(N_{i}\right)\right)=\frac{1}{\left(\mathrm{FDDx}\;\mathrm{RDD}\right)}$

  c.
Rank (Aggi)= Rank(PN)+ Rank_Inc
  d.
$\mathrm{Rank}\_\mathrm{Inc}\;=\;\mathrm{ETX}\;\left(\mathrm{Agg}\left(N_{i}\right)\right)+\;P\left({\mathrm{Agg}}_{i}\right)+\;\mathrm{Min}\_\mathrm{hop}\_\mathrm{rank}\_\mathrm{increase}$
  e.
**If** 
Best_Aggregator >= Preferred_Aggregator
**Then** Best_Aggregator = Preferred_Aggregator  **f. End** **2. End** **3. While** Preferred_Aggregator = Best_Aggregator **do**  a.
Candidate_Aggregator_Parent_ID = Preferred_Aggregator
 **4. End** **5. Return Optimal Aggregator parent**

**Figure 2:**
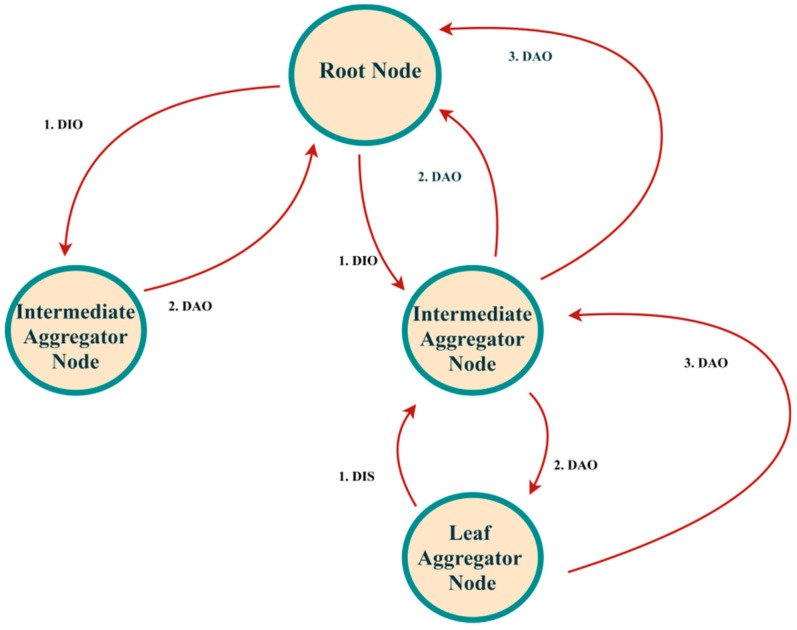
DODAG formation process.

Figure 2 represents the simple scenario that a network consists of three nodes and a root with the formation of DODAG towards the root. The steps are listed below.

1. DODAG_ROOT sends DODAG Information object (DIO) to AGG_A;
2. AGG_A replies Destination Advertisement Object (DAO) to DODAG_ROOT;
3. AGG_A sends DIO to AGG_B;
4. AGG_B replies DAO to AGG_A;
5. AGG_B sends DIO to AGG_C;
6. AGG_C replies DIO to AGG_B;
7. Completing all the ICMPV6 messages, DODAG root nodes know all the prefix information of the route towards the leaf nodes.

#### Expected Transmission Count

Expected transmission count [32] is a metric to identify the path quality given by Equation (5). It is calculated from forward data delivery (FDD) to reverse data delivery (RDD).

(5) $$\mathrm{ETX}\;\left(\mathrm{Agg}\left(N_{i}\right)\right)=\frac{1}{\left(\mathrm{FDD}*\;\mathrm{RDD}\right)}$$

Power Factor: The power factor of the aggregator node is derived from residual energy. The power factor is calculated from Equation (6).

(6) $$P\left({\mathrm{Agg}}_{i}\right)\;=\frac{\left(\mathrm{Initial}\_\mathrm{power}\;–\;\mathrm{Consumed}\_\mathrm{power}\;\right)\;}{\mathrm{Initial}\_\mathrm{power}\;}$$

Objective Function: The objective function acts as the main point to select the parent for the DODAG formation and optimize the route towards the ROOT node. The proposed metric combines ETX and the power factor of the node to enhance the efficiency of the RPL protocol. The objective functions used by the proposed work are as follows:

- ETX objective function.
- Power factor.

**Figure 3:**
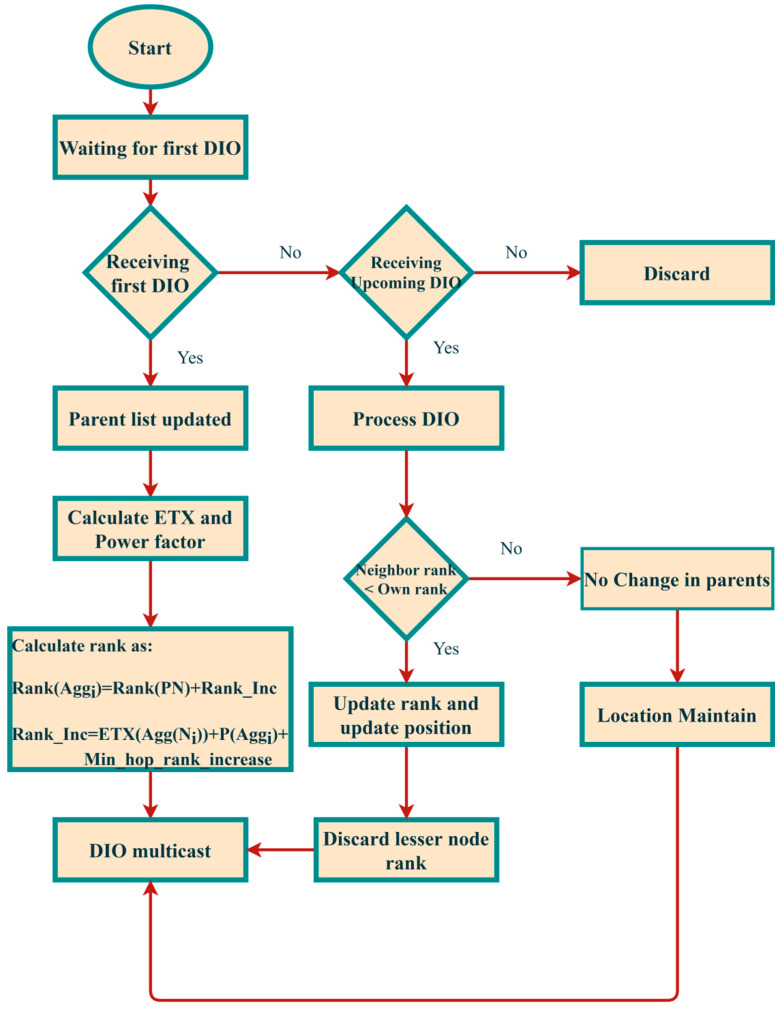
Parent selection based on ETX and energy.

Rank Calculation: The rank calculates parent rank and rank increase. The rank increase is calculated from step and MinHopRankIncrease, where the MinHopRankIncrease default value is 256 [33]. The step value is calculated from the objective function, and it returns the scalar value. The rank calculation formula is denoted in Equations (7) and (8). Parent selection using rank metric is explained in Figure 3.

(7) $$\;\mathrm{Rank}\;({\mathrm{Agg}}_{i})\;=\mathrm{Rank}\left(\mathrm{PN}\right)+\mathrm{Rank}\_\mathrm{Inc}$$

(8) $$\mathrm{Rank}\_\mathrm{Inc}\;=\;\mathrm{ETX}\;\left(\mathrm{Agg}\left(N_{i}\right)\right)+\;P({\mathrm{Agg}}_{i})+\;\mathrm{Min}\_\mathrm{hop}\_\mathrm{rank}\_\mathrm{increase}$$

## 5. Performance Evaluation

### 5.1. Simulation Setup

The performance evaluation of the proposed CAA-ERPL protocol against energy-efficient RPL(E-RPL) is done using a Contiki Cooja simulator [16,34]. Sky motes are taken for the entire simulation. Sky motes have a 16-bit MSP430 MCU, 10 kB RAM, 48 kB ROM, a cc2420 802.15.4 radio transceiver, an external Flash memory, and a temperature, humidity, and brightness sensor [34]. Sensor nodes are located within the transmission range of every aggregator that comes under one region. The forest area is divided into multiple regions of unequal size. The aggregator is responsible for collecting and aggregating the data for forwarding to border router. The data are transmitted periodically from the sensor nodes every 60 s. The simulation is done by varying the number of nodes in the network as 10, 20, 30, 40, and 50. The simulation parameters are listed in Table 1.

### 5.2. Performance Metrics

Packet Delivery Ratio (PDR): PDR is used to identify whether all the data packets sent from the sensor nodes reached the sink or not. PD represents the rate at which the data reach the sink node. For successful communication, PDR is 100%. PDR is calculated simply from Equation (9).

(9) $$\mathrm{PDR}\;=\frac{\;\mathrm{TPR}}{\mathrm{TPS}\times 100}\;$$

$$\begin{array}{c} \mathrm{TPR}—\mathrm{Total}\;\mathrm{number}\;\mathrm{packets}\;\mathrm{received}\\ \mathrm{TPS}—\mathrm{Total}\;\mathrm{number}\;\mathrm{packets}\;\mathrm{sent} \end{array}$$

Latency: Latency is calculated based on the congestion in the network. It is the time taken between the time of the initial data packet sent (*T_0_*) and the time of the last acknowledgment received from the sink (*T_n_*). Latency is computed from Equation (10).

(10) $$\mathrm{Latency}\;\mathrm{Delay}\;\left(s\right)=\frac{\;\left(T_{n}\;–T_{0}\right)\times 100}{2\;\times 128}$$

Power Consumption (PC): For forest networks, all the sensor nodes are non-rechargeable. The power of the node decides the lifetime of the node. In the case of E-RPL, the energy of the node in both the CPU and LPM mode is taken. While calculating the energy consumption, both the mode powers are considered along with transmitting and listening power and are expressed in Equation (11). Average power consumption is calculated for all the nodes in the network and given by Equation (12).

(11) $$\mathrm{Total}\;\mathrm{Power}\;\mathrm{Consumption}=\sum_{j=1}^{\mathrm{sn}}\left(P_{\mathrm{LPM}}+P_{\mathrm{CPU}}+P_{\mathrm{RL}}+P_{\mathrm{RT}}\right)$$

$$\begin{array}{c} P_{\mathrm{LPM}}=\mathrm{Amount}\;\mathrm{of}\;\mathrm{power}\;\mathrm{consumption}\;\mathrm{during}\;\mathrm{low}\;\mathrm{power}\;\mathrm{mode}\\ P_{\mathrm{CPU}}=\mathrm{Amount}\;\mathrm{of}\;\mathrm{power}\;\mathrm{consumption}\;\mathrm{during}\;\mathrm{normal}\;\mathrm{CPU}\;\mathrm{mode}\\ P_{\mathrm{RL}}=\mathrm{Amount}\;\mathrm{of}\;\mathrm{power}\;\mathrm{consumed}\;\mathrm{for}\;\mathrm{radio}\;\mathrm{listening}\\ P_{\mathrm{RT}}=\mathrm{Amount}\;\mathrm{of}\;\mathrm{power}\;\mathrm{consumed}\;\mathrm{for}\;\mathrm{radio}\;\mathrm{transmission}\\ \mathrm{sn}=\mathrm{Total}\;\mathrm{number}\;\mathrm{of}\;\mathrm{sensor}\;\mathrm{nodes}\;\mathrm{in}\;\mathrm{the}\;\mathrm{network} \end{array}$$

(12) $$\mathrm{Average}\;\mathrm{Power}\;\mathrm{Consumption}=\frac{\sum_{j=1}^{\mathrm{sn}}\left(P_{\mathrm{LPM}}+P_{\mathrm{CPU}}+P_{\mathrm{RL}}+P_{\mathrm{RT}}\right)}{\mathrm{sn}}$$

### 5.3. Result Analysis

#### 5.3.1. Power Consumption

**Figure 4:**
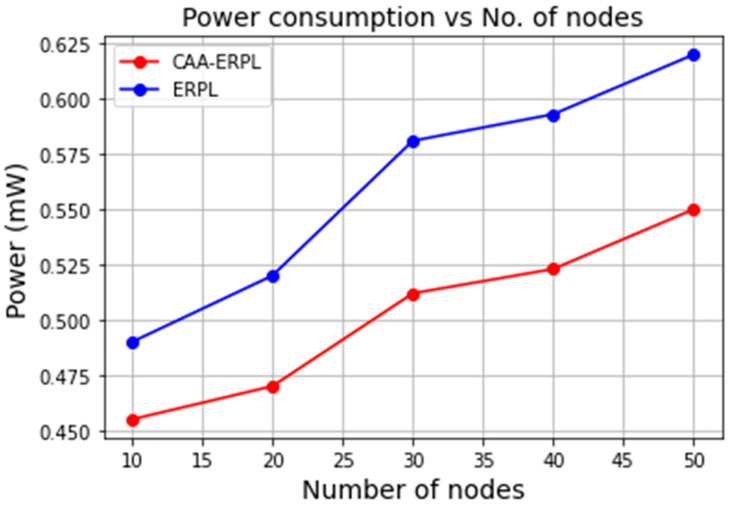
Total power consumption vs. scalability of nodes.

Figure 4 shows the power consumption of the entire network by varying the number of nodes in the network for CAA-ERPL versus E-RPL. It is seen that the power consumption of CAA-ERPL decreases with an increasing number of nodes as compared to E-RPL. The reason for such a decrease in power consumption in CAA-ERPL is that aggregators are defined based on the number of nodes. In E-RPL, all sensor nodes form DODAG based on an objective function, which is residual power for communicating to the border router. So, as the number of nodes increases, the sensor nodes will consume more power for communicating to the border router, which is far away. In CAA-ERPL, the aggregator is formed based on the number of nodes in the region for aggregating and compressing the data for transmission to the border router. This ultimately reduces the amount of power dissipated by sensor nodes. Aggregators play a vital role in aggregating and compressing the sensor data for communication with the border router. This can be achieved by forming DODAG with less power consumption and reduced communication.

#### 5.3.2. Average Hop Count

**Figure 5:**
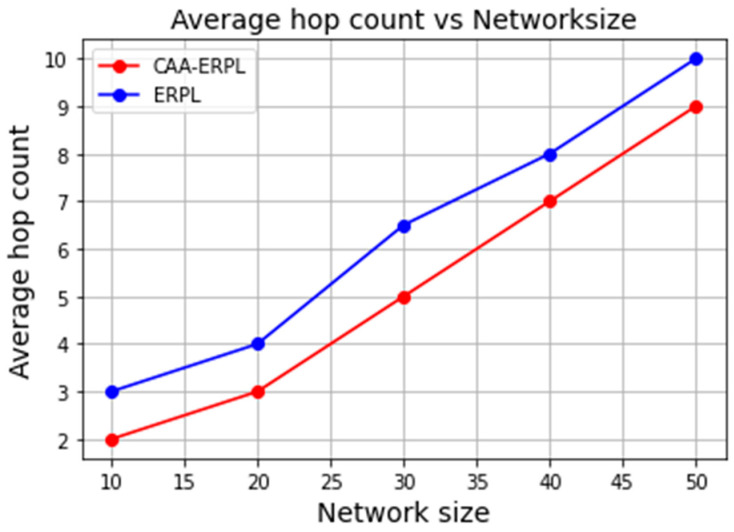
Average hop count vs. network size.

When a dense network is considered, the number of nodes is high and is located near each other. More nodes are deployed in the small forest region. To perform DODAG for all the nodes in the network, it is efficient to do routing using RPL only for aggregators. The average number of nodes in the DODAG formation towards the root varies for both cases. Figure 5 represents the average number of hop count in the final DODAG from source to DODAG ROOT for both protocols. The average hop count of CAA-ERPL is 2, 3, 5, 7, 9 and, for E-RPL, is 3, 4, 6.5, 8, 10. In CAA-ERPL, the average hop count decreases, as it processes first data aggregation and then routing. So, it provides the stability in the network, though it is dense.

#### 5.3.3. Overhead

**Figure 6:**
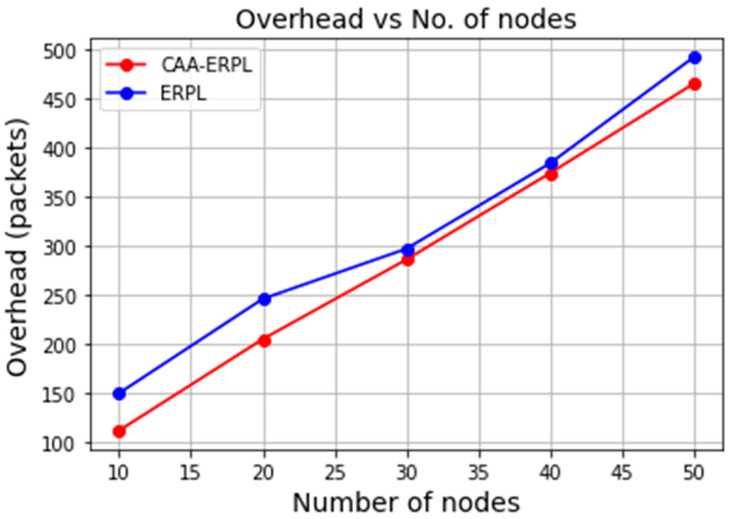
Overhead vs. number of nodes.

Overhead depicts the number of packets involved in the transmission. The proposed CAA-ERPL is compared with the E-RPL in terms of overhead. It is found that for both protocols, the overhead increases when the network size increases. As we are involving the aggregator and more data packets sent from sensor nodes are compressed and forwarded towards the root node, the number of packets involved in the transmission is less when compared to E-RPL. From Figure 6, it is seen that the overhead of CAA-ERPL and E-RPL is 205 and 246, respectively, for the network size of 20 nodes.

#### 5.3.4. Packet Delivery Ratio

**Figure 7:**
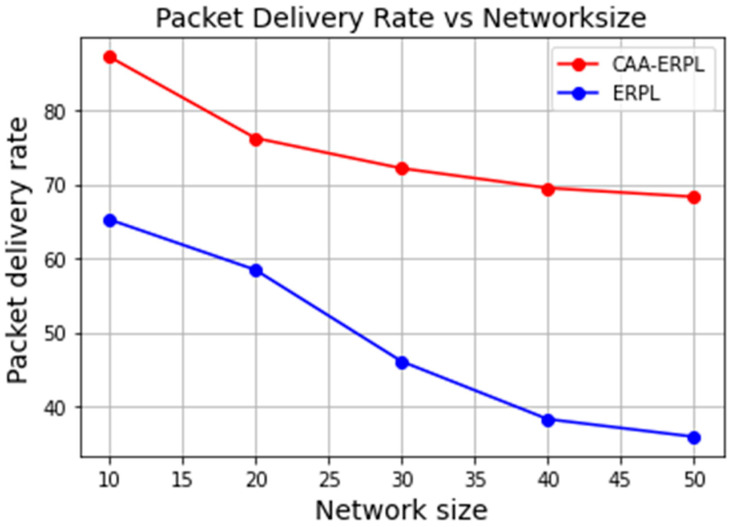
PDR vs. scalability.

Generally, the packet delivery ratio decreases with an increase in the number of nodes. The packet loss ratio is inversely proportional to PDR. When the nodes are increased in the network and randomly deployed with partial nodes in the interference range, it will take more time to communicate to the sink node. From Figure 7, it is understood that PDR decreases with the increasing number of nodes. In E-RPL, drastically, the PDR reduced from 60% to 40% when the number of nodes increased from 20 to 40, as nodes are in the interference range, and also, the farthest node would take more time to communicate to the border router, resulting in increased delay and packet loss.

In the proposed CAA-ERPL, because of the aggregation, there is no drastic decrease in the PDR. The reason being that the formation of an aggregator for sensor nodes in the region is responsible for aggregating and compressing the data, thereby forming DODAG with a border router for communication. This ultimately increases the packet delivery ratio, as aggregators are involved in DODAG formation with the border router for data communication. Additionally, aggregators are formed for a small number of nodes in the region where there would be no packet loss. That is the reason the PDR of CAA-ERPL is 68% to 89% for varying levels of nodes in the network as compared to E-RPL.

#### 5.3.5. Average Number of Parent Changes

**Figure 8:**
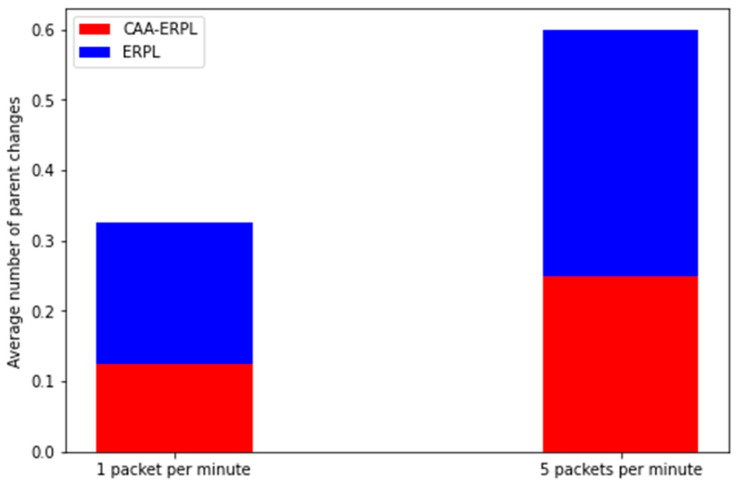
Average number of parent changes vs. data rate.

It specifies the stability of the network throughout the simulation time. Figure 8 illustrates the average number of parent changes in the proposed and existing RPL protocols. Two different scenarios of execution are done by varying the rate of transmission. One packet/min and 5 packets/min are the two cases taken for simulation. From Figure 8, it is observed that the average parent changes values are 0.12 and 0.32 for CAA-ERPL and E-RPL, respectively. The average number of parent changes is low when compared to the E-RPL. The reason behind this is aggregation in the network. Only the aggregator nodes participate in the DODAG, and henceforth, possibilities will be low for changing the parent frequently. From Figure 8, it is inferred that for the simulation of 5 packets/min, the average parent change is 0.25 and 0.6 for CAA-ERPL and ERPL, respectively.

#### 5.3.6. Average Delay

**Figure 9:**
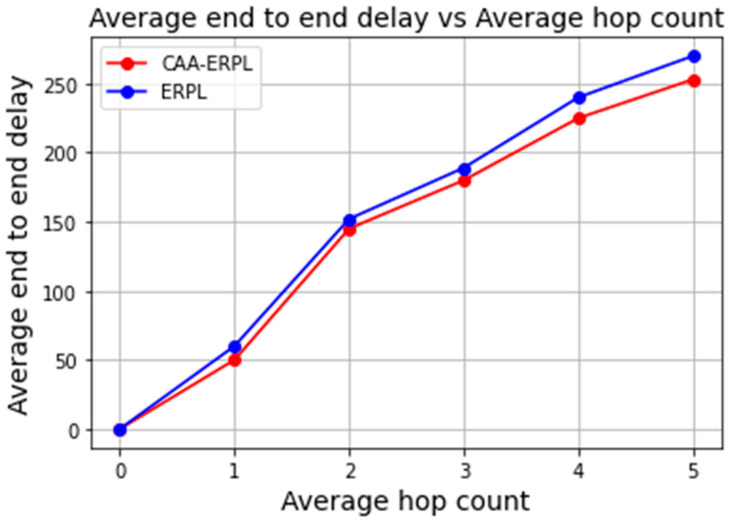
Average end-to-end delay vs. average hop count.

This represents the total time taken for the data transmission from the source node to the border router. With respect to the hop count of the nodes from the root, the delay is calculated in milliseconds. For the data rate of 1 packet/min, the end-to-end delay values are 250 and 270 ms for CAA-ERPL and E-RPL, respectively, for the maximum hop count of 5. Generally, LLN consumes more time for data transmission because of its constrained resources. Average end-to-end delay acts as one of the major metrics that represent the timely delivery of data. In CAA-ERPL, the aggregator node routes the aggregated and compressed data to the border router by forming DODAG, which reduces the number of transmissions to the border router. In E-RPL, all nodes participate in forming the DODAG with the border router for sending. This ultimately results in an increased delay for E-RPL in sending the data to the border router as compared to CAA-ERPL, where aggregator forms DODAG with a border router in routing the aggregated and compressed data, as shown in Figure 9.

#### 5.3.7. CPU Power Utilization

**Figure 10:**
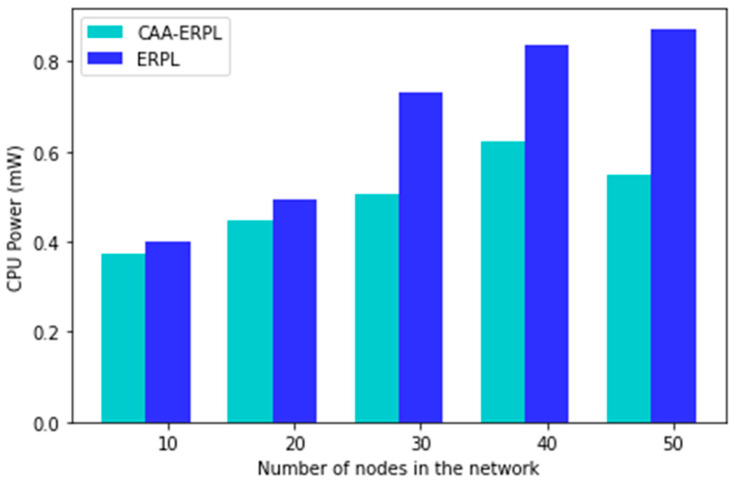
CPU utilization vs. scalability of the network.

Figure 10 shows the CPU utilization of the entire simulation with a time period of 45 min. As the number of nodes increases, the CPU utilization increases. The average CPU power consumption of the entire network in the proposed CAA-ERPL is 0.07%, 0.10%, 0.31%, 0.26%, and 0.37% when compared to E-RPL. This is because the routing is formed for the aggregators only. The compressed data are sent via the DODAG parent to reach the border router.

### 5.4. Overall Result Discussion

The proposed work is evaluated with sky motes by considering the metrics such as total power consumption, delay, frequent number of parent changes in routing, packet delivery ratio, etc. It is highly important to analyze the working of RPL by varying the application scenarios. Here, the IoT–Fog-based forest environment is taken for analysis. The proposed CAA-ERPL protocol improved the features of RPL by adding energy as an objective function and achieved a solution to the load imbalance problem by routing through region-based aggregators. The experiments were done with various network sizes such as 10, 20, 30, 40, and 50 nodes. It is seen that the power consumption and average CPU power consumption of CAA-ERPL decrease with an increasing number of nodes as compared to E-RPL. In CAA-ERPL, the average hop count decreases, as it processes first data aggregation and then routing. Stability in the network is maintained for CAA-RPL with varying network size as compared to E-RPL. The overhead of CAA-ERPL and E-RPL is 500 and 460, respectively, for the network size of 50 nodes. The PDR of CAA-ERPL is 70%, whereas for E-RPL, it is 20% for 50 nodes setup. When the end-to-end delay is considered for the performance, it increases maximum up to 250 ms in CAA-ERPL, whereas, it is 280 ms in E-RPL. Lastly, the average number of parent changes is low in CAA-ERPL when compared to the E-RPL. In summary, the overall comparison of the proposed CAA-ERPL minimized a large number of control overheads, thereby increasing the PDR, reducing the data packet delay in the network with efficient energy consumption.

## 6. Conclusions and Future Works

Forest management is very much needed to improve environmental sustainability and maintain the biological balance in the world. Much real-time research is going on for better communication in the forest region with the evolution of IoT. Smart forest management is done with the deployment of sensor nodes and communication among them, which will take more energy. Since the nodes are battery powered, there are possibilities for energy constraints in remote monitoring. To extend the lifetime of the network, aggregation and data compression along with routing are proposed in CAA-ERPL. The proposed CAA-ERPL has three phases. First, based on the transmission range, aggregator nodes are chosen for each forest region. Next, each region node communicates with its aggregator. Finally, routing based on RPL is framed by using ETX and energy as objective functions for all aggregators. The performance of the proposed CAA-ERPL protocol is compared with E-RPL in Contiki Cooja based on metrics such as overhead, end-to-end delay, CPU power utilization, number of parent changes in DODAG, and PDR. From the analysis, it has been observed that the proposed CAA-ERPL is better than E-RPL.

In the future, the work can be extended to implement various real-time sensors related to forest monitoring and communicating the data packets using aggregator nodes via energy-based RPL to validate the performance. To improve the performance of the sensor nodes and the lifetime of the network, optimization can be done in the Fog node or border router placement that will help to communicate and process the intermediate real-time data. Lastly, the fuzzy-based approach can be integrated by choosing the best residual power and ETX objective function in CAA-ERPL towards forming the DODAG for effective performance.

## Figures and Tables

**Table 1:** Simulation setup.

Parameter	Range of Value
OS	Instant Contiki 3.0
Network area	500 m × 500 m
Mote type	Sky mote
MAC	802.15.4
Interval time for transmission	60 s
Propagation model	Unit disk graph
Time of simulation	45 min
Data transmission Interval	1 min
RPL objective function	ETX, Power
Topology	Hierarchical
Number of nodes	10, 20, 30, 40, 50

## Data Availability

Not applicable.
